# The association between upper limb function, physical exercise, and cognitive ability among empty-nest elderly in China: A cross-sectional study based on CLHLS

**DOI:** 10.1371/journal.pone.0351211

**Published:** 2026-06-10

**Authors:** Quan Zhou, Jiaran Jiang, Chuanxia Zhang, Yiming Ma

**Affiliations:** 1 Department of General Education, Shandong Xiehe University, Jinan, Shandong, China; 2 Graduate School of Arts and Science, Gachon University, Seongnam, Gyeonggido, Republic of Korea; ISSEP Kef: Universite de Jendouba Institut Superieur du Sport et de l'Education Physique du Kef, TUNISIA

## Abstract

**Background:**

The rapid aging of China’s population poses serious challenges to the cognitive health of older adults living without co-resident children (empty-nest older adults). Currently, the role of upper limb function on the cognitive function of this group and its potential pathway of effect through physical exercise remain unclear.

**Objective:**

This study aimed to explore the associations among upper limb function, physical exercise, and cognitive ability in the Chinese empty-nest elderly population, and to explore the potential mediating role of physical exercise in the relationship between upper limb function and cognition. Due to the cross-sectional design, no causal relationships can be established; all reported associations are statistical in nature.

**Methods:**

This study employed a cross-sectional design using data from the 2018 Chinese Longitudinal Healthy Longevity Survey (CLHLS). Based on household structure information, older adults aged 60 and above who did not live with their children were defined as “empty-nest elderly”. A total of 5,060 empty-nest elderly were included in the final analysis. Upper limb function (normal/restricted), physical exercise (yes/no), and cognitive ability (normal/impaired) were assessed via questionnaire. Analyses included multivariable logistic regression, restricted cubic spline curves, Bootstrap-based mediation effect analysis, and subgroup analysis.

**Results:**

Among the 5,060 participants (mean age 78.60 ± 10.31 years), 755 (14.92%) had cognitive impairment. Multivariable-adjusted analysis showed that restricted upper limb function was significantly positively associated with the risk of cognitive impairment (OR=2.55, 95% CI: 1.95–3.29), while regular physical exercise was significantly negatively associated with this risk (OR=0.77, 95% CI: 0.62–0.95). Mediation analysis indicated that physical exercise accounted for 5.95% of the total effect (ACME = 0.0121, 95% CI: 0.0074–0.0172). Given the small effect size, this pathway should be interpreted as a minor statistical contributor rather than a dominant mechanism. Subgroup analysis showed that the association of upper limb function and physical exercise with cognitive impairment was significantly modified by drinking status (both P for interaction < 0.05), with stronger effects in non-drinkers.

**Conclusion:**

Among Chinese empty-nest elderly, restricted upper limb function is positively associated with the risk of cognitive impairment, while regular physical exercise is negatively associated with this risk. Physical exercise accounts for a minor portion of the statistical association between upper limb function and cognitive ability.

## Introduction

China’s population aging process has been accelerating continuously since the late 1970s, with an average annual growth rate of about 3.2% [1]. By 2020, the population aged 60 and above accounted for 18% of the total population, with those aged 80 and above making up 2.5%; it is projected that by 2050, this proportion will rise to 39%, and the population aged 80 and above will account for 10% [2]. This structural change has directly increased the overall size of the empty-nest elderly population. Consequently, the healthcare burden has intensified. Particularly under the promotion of the Diagnosis-Related Groups (DRGs) payment model, treatment costs for chronic diseases have increased significantly. Although medication expenditures have been somewhat controlled, overall healthcare economic pressure continues to rise [3]. Regarding mental health, loneliness is prevalent among older adults living alone, with migrant older adults facing even higher risks [4]. The incidence of depressive symptoms among older adults in nursing institutions also reaches 36.8% [5]. Furthermore, loneliness is significantly associated with decreased sleep quality, and insufficient social support exacerbates this phenomenon [6].

In addition to mental health issues, the maintenance of physical function, especially upper limb function, is crucial for the independent living of older adults. Relevant studies indicate that impaired upper limb motor function directly limits the ability of older adults to perform basic activities of daily living (ADL) such as dressing, eating, and washing [7]. Notably, this functional impairment stems not only from objective motor deficits but is also closely related to the individual’s subjective perception of their own motor ability. For instance, chronic stroke survivors often underestimate their actual upper limb motor ability, and this underestimation is not necessarily correlated with the degree of impairment itself [8]. Further research shows that performance characteristics of upper limb motor function (such as slowed speed, increased errors, and greater movement variability) are significantly associated with cognitive impairment (including mild cognitive impairment and subjective cognitive decline) [9]. These abnormal movement features often accompany cognitive impairment, making them a potential biomarker for distinguishing normal aging from pathological cognitive decline.

Exercise intervention has been widely recognized as an effective strategy for improving cognitive function in older adults. Research indicates that all forms of physical exercise significantly improve cognitive function, but mind-body exercise is considered the optimal intervention, demonstrating superior cognitive benefits, adherence, and related functional advantages [10]. For patients with cognitive impairment, especially dementia, resistance exercise is most likely to be the best type of exercise for slowing cognitive decline. Multicomponent exercise is most effective in protecting overall cognition and executive function in patients with mild cognitive impairment (MCI) [11].

As a special subgroup within the older adult population, empty-nest elderly face unique health challenges. Although existing evidence supports the effectiveness of exercise interventions in improving cognitive function in older adults, research specifically focusing on the association between upper limb function and cognitive function in the Chinese empty-nest elderly population remains relatively limited. Most existing studies focus on lower limb function or overall physical activity, lacking in-depth exploration of the specific role of upper limb function in cognitive aging. Furthermore, due to their relatively weak social support systems, barriers to participating in regular exercise for empty-nest elderly differ from those of the general older adult population, necessitating targeted intervention strategies. This study aims to fill this knowledge gap by exploring the associations among upper limb function, physical exercise, and cognitive ability in Chinese empty-nest elderly, providing a scientific basis for developing early identification and exercise intervention strategies for cognitive impairment in this specific population. Against the backdrop of continuing population aging, identifying low-cost, high-benefit strategies for preventing cognitive decline holds significant public health importance. By clarifying the association between upper limb function and cognitive function, and the role mechanism of physical exercise therein, this study is expected to provide empirical support for promoting healthy aging among Chinese empty-nest elderly. Based on neurobiological models suggesting that physical activity enhances cognitive reserve through neurogenesis and synaptic plasticity, and that upper limb function may influence the capacity to engage in such activity, we hypothesized that physical exercise might statistically mediate the association between upper limb function and cognitive ability. However, due to the cross-sectional design, this hypothesized pathway should be interpreted as a statistical association, not a causal mechanism.

## Materials and methods

### Data source

This study employed a cross-sectional design, analyzing data from the 2018 Chinese Longitudinal Healthy Longevity Survey (CLHLS). The CLHLS is a nationally representative survey of the middle-aged and older adult population in China, collecting extensive information on health status, physical function, cognitive performance, lifestyle behaviors, and socioeconomic factors.

### Population selection

**Fig 1:**
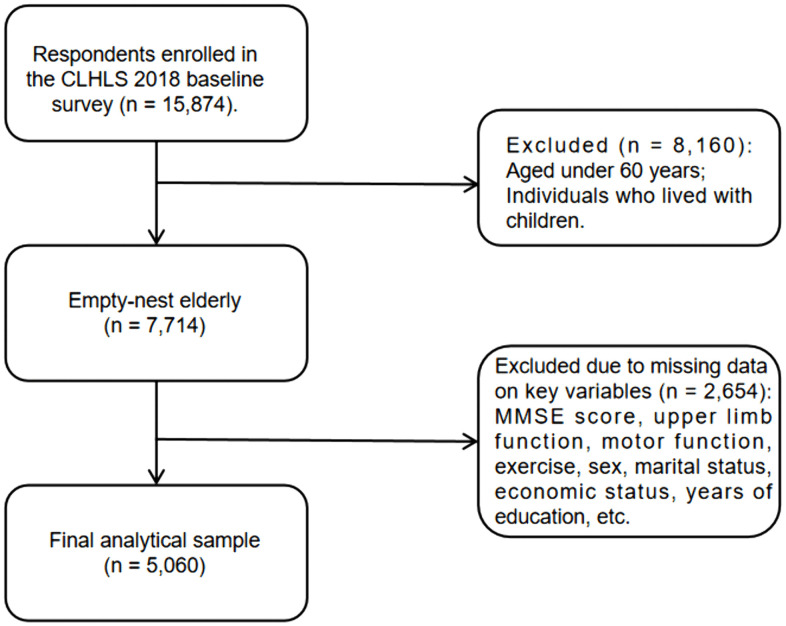
Sample selection flowchart.

The initial sample of this study consisted of 15,874 cases. According to the inclusion and exclusion criteria, a total of 7,714 participants were first identified as meeting the definition of empty-nest elderly. Among these, 2,654 cases were excluded due to missing data on key variables (such as MMSE score, upper limb function, motor function, etc.), resulting in a final analytical sample of 5,060 cases, as illustrated in Fig 1. To assess potential selection bias caused by missing data, this study compared the baseline characteristics of the included participants (n = 5,060) with those excluded due to missing data (n = 2,654) (S1 Table). The results showed no statistically significant differences between the two groups in the majority of characteristics (P > 0.05).

### Variable selection and assessment

Upper Limb Function was assessed through three functional movements in the “G8” section of the CLHLS questionnaire: G8-1 Hand to back of neck, G8-2 Hand to small of back, G8-3 Raise arm overhead. The completion status for each movement had the following four options: 1=”Right hand only”, 2 = ”Left hand only”, 3 = ”Both hands”, 4 = ”Neither hand”. Each item was scored as follows: “Both hands” = 3 points, “One hand only” = 2 points, “Neither hand” = 1 point. The total score ranged from 3 to 9, with higher scores indicating better upper limb function. We defined individuals who achieved the “Both hands” standard in all three movements as having “Normal upper limb function”.

Physical Exercise was assessed based on the question in the CLHLS regarding physical exercise: “Do you regularly engage in physical exercise?”, with answer options “Yes” or “No”. Participants answering “Yes” were categorized as “Engaged in physical exercise”, and those answering “No” as “Not engaged in physical exercise”.

Cognitive Function was assessed using the cognitive function module in the CLHLS, which typically includes orientation (awareness of time, place, person), memory (immediate and delayed recall), attention and calculation ability (e.g., simple mental arithmetic tests). The score ranged from 0 to 31, with higher scores indicating better cognitive performance. For regression analysis, the raw cognitive scores were converted to Z-scores by subtracting the mean and dividing by the baseline standard deviation. Negative Z-values indicated poorer cognition and were classified into the cognitive impairment group [12].

Based on literature review, this study included the following covariates: Demographic characteristics: Age (continuous), Gender (male/female), Marital status (married/other). Socioeconomic status: Years of education (continuous), Self-rated economic status (affluent and above/average and below). Health-related behaviors: Smoking history (yes/no), Drinking history (yes/no).

### Ethical statement

The CLHLS study was approved by research ethics committees of Duke University and Peking University (IRB00001052–13074). All participants provided written informed consent. No experimental interventions were performed. An exempted Institutional Review Board (IRB) protocol was approved by Duke University (Pro00062871).

### Statistical analysis

Given the cross-sectional nature of the data, causal inferences are not possible. All statistical analyses were performed using R software (version 4.4.3), with the statistical significance level set at α = 0.05 (two-tailed). First, descriptive analysis was conducted for all variables included in the study. Categorical variables were presented as frequency (n) and percentage (%), shown for the total sample and subgroups stratified by cognitive status (normal vs. impaired), with between-group comparisons using the chi-square test. Continuous variables were described using mean ± standard deviation (Mean ± SD). For all continuous variables in the baseline table, between-group comparisons were conducted using the Independent Samples t-test to examine if there were statistically significant differences between groups (e.g., cognitively normal group vs. cognitively impaired group). To explore the potential mediating role of physical exercise in the statistical association between upper limb function and cognitive status, we employed a regression-based mediation analysis framework (note: causal interpretation is not justified given the cross-sectional design). Specifically, first, a logistic regression model (mediator model) was fitted with physical exercise (binary variable) as the outcome variable, upper limb function (categorical independent variable) as the predictor, adjusting for all covariates. Model 1 was unadjusted. Model 2 was adjusted for demographic characteristics (age, gender, marital status). Model 3 was further adjusted for years of education, self-rated economic status, smoking, drinking, and residence (fully adjusted model). Subsequently, a logistic regression model (outcome model) was fitted with cognitive status (binary variable) as the outcome variable, simultaneously including upper limb function (direct effect) and physical exercise (mediating effect) as predictors, adjusting for the same covariates. We used the nonparametric Bootstrap method with 1000 resamples to estimate the Average Causal Mediation Effect (ACME), Average Direct Effect (ADE), and Total Effect, and calculated their 95% confidence intervals. The proportion mediated was calculated as the ratio of ACME to the Total Effect. If the 95% CI for ACME did not include 0, the mediating effect was considered statistically significant. Furthermore, to assess the independence and robustness of the association between upper limb function and cognitive impairment, we constructed a multivariable logistic regression model adjusting for all covariates, calculating the odds ratio (OR) and 95% confidence interval (CI). Stratified analysis was further conducted by gender, marital status, economic status, smoking, drinking, and residence. Effect modification was explored by including interaction terms (e.g., upper limb function × gender) in the regression models for each stratum and calculating the p-value for interaction using the likelihood ratio test. Sensitivity analysis was performed using linear regression.

## Results

As shown in Table 1, the study included 5,060 empty-nest elderly (mean age 78.60 ± 10.31 years, 42.57% male), divided into a cognitively normal group (4,305 individuals) and a cognitively impaired group (755 individuals) based on cognitive status. Compared to the cognitively normal group, participants in the cognitively impaired group exhibited the following significant characteristics: older age, higher proportion of males, lower proportion of married status, fewer average years of education, lower proportion self-rating economic status as “affluent and above”, less smoking and drinking behavior, significantly less physical exercise behavior, more prevalent upper limb function restriction, and a higher proportion residing in towns and rural areas.

**Table 1:** Baseline Characteristics of Participants Grouped by Cognitive Status.

Variables	Total (n = 5,060)	Normal cognition (n = 4,305)	Cognitive impairment (n = 755)	P
**Age, Mean±SD**	78.60 ± 10.31	76.70 ± 9.03	89.41 ± 10.51	<0.001
**Years of Education, Mean±SD**	4.74 ± 4.69	5.26 ± 4.69	1.78 ± 3.45	<0.001
**Gender, n(%)**				<0.001
Male	2154 (42.57)	1,729 (40.16)	425 (56.29)	
Female	2906 (57.43)	2,576 (59.84)	330 (43.71)	
**Marry, n(%)**				<0.001
Others	689 (13.62)	384 (8.92)	305 (40.40)	
Married	4,371 (86.38)	3,921 (91.08)	450 (59.60)	
**Economic Status, n(%)**				<0.001
Average and below	4,017 (79.39)	3,367 (78.21)	650 (86.09)	
Affluent and above	1,043 (20.61)	938 (21.79)	105 (13.91)	
**Smoke**, n(%)				<0.001
No	4,075 (80.53)	3,420 (79.44)	655 (86.75)	
Yes	985 (19.47)	885 (20.56)	100 (13.25)	
**Drink, n(%)**				<0.001
No	4,116 (81.34)	3,449 (80.12)	667 (88.34)	
Yes	944 (18.66)	856 (19.88)	88 (11.66)	
**Exercise, n(%)**				<0.001
No	3,101 (61.28)	2,509 (58.28)	592 (78.41)	
Yes	1,959 (38.72)	1,796 (41.72)	163 (21.59)	
**Upper Limb Function, n(%)**				<0.001
Normal	4,590 (90.71)	3,992 (92.73)	598 (79.21)	
Restricted	470 (9.29)	313 (7.27)	157 (20.79)	
**Residence, n(%)**				<0.001
City	1,316 (26.01)	1,176 (27.32)	140 (18.54)	
Town	1,612 (31.86)	1,350 (31.36)	262 (34.70)	
Rural	2,132 (42.13)	1,779 (41.32)	353 (46.75)	

**Fig 2:**
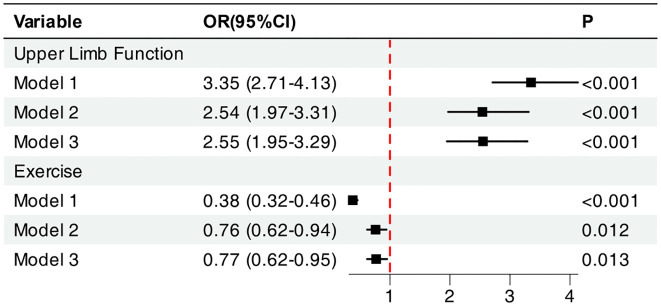
Forest plot showing the association of upper limb function and regular exercise with cognitive impairment.

As shown in Fig 2, multivariable logistic regression models were used to assess the association of upper limb function and physical exercise with the risk of cognitive impairment. Across three models with stepwise adjustment for confounders, restricted upper limb function was significantly positively associated with the risk of cognitive impairment, while physical exercise was significantly negatively associated with this risk. These associations remained statistically significant after adjusting for different sets of confounders. Specifically, for restricted upper limb function: in Model 1 (unadjusted), OR=3.35 (95% CI: 2.71–4.13, p < 0.001); in Model 2 (adjusted for some confounders), OR=2.54 (95% CI: 1.97–3.31, p < 0.001); in Model 3 (further adjusted for more confounders), OR=2.55 (95% CI: 1.95–3.29, p < 0.001). For physical exercise: in Model 1 (unadjusted), OR=0.38 (95% CI: 0.32–0.46, p < 0.001); in Model 2, OR=0.76 (95% CI: 0.62–0.94, p = 0.012); in Model 3, OR=0.77 (95% CI: 0.62–0.95, p = 0.013).

**Fig 3:**
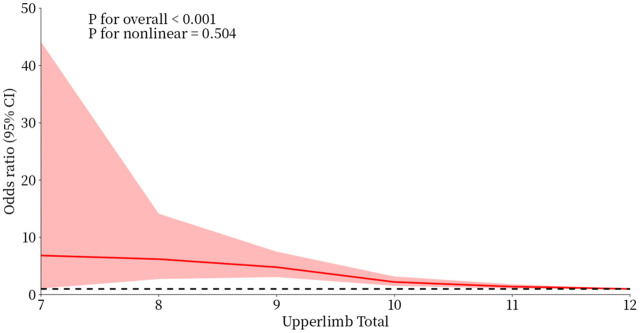
Association between upper limb function and cognitive impairment analyzed by restricted cubic splines.

We used restricted cubic spline models to explore potential nonlinear associations between upper limb function and the risk of cognitive impairment. As shown in Fig 3, the association between the two was significant (p < 0.001), and no significant nonlinear relationship was found (P for nonlinear = 0.504). The analysis results showed that as upper limb function improved, the risk of cognitive impairment generally showed a downward trend.

**Fig 4:**
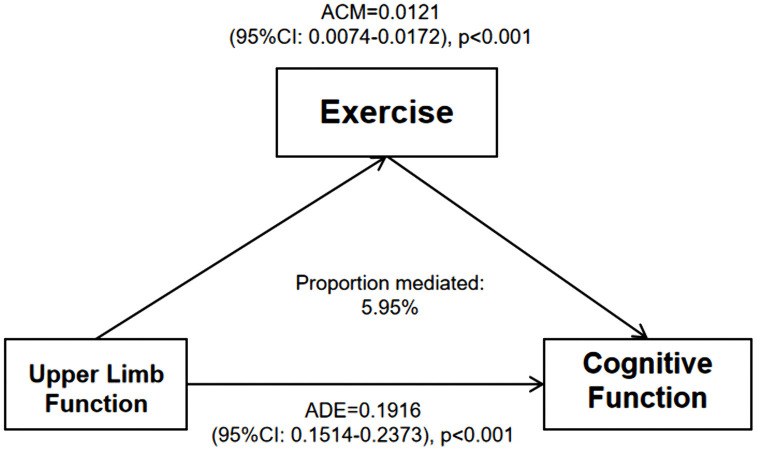
Mediation analysis of the association between upper limb function and cognitive function through physical exercise.

As shown in Table 2 and Fig 4, after adjusting for covariates including age, gender, marital status, years of education, economic status, smoking, and drinking, the mediation analysis indicated a significant mediating effect of the mediator variable (Average Causal Mediation Effect ACME = 0.0121, 95% CI: 0.0074–0.0172, p < 0.001), while the direct effect was also significant (Average Direct Effect ADE = 0.1916, 95% CI: 0.1514–0.2373, p < 0.001). The mediation effect accounted for approximately 5.95% of the total effect.

**Table 2:** Mediation analysis of the effect of upper limb function on cognitive impairment through exercise.

Variable	Estimate	95% CI Lower	95% CI Upper	p-value
**Average Causal Mediation Effect (ACME)**	0.0121	0.0074	0.0172	<0.001
**Average Direct Effect (ADE)**	0.1916	0.1514	0.2373	<0.001
**Total Effect**	0.2038	0.1616	0.2504	<0.001
**Proportion Mediated (Average)**	0.0595	0.0359	0.0843	<0.001

As shown in Table 3, restricted upper limb function was significantly positively associated with the risk of cognitive impairment (OR=2.55, 95% CI: 1.95–3.29, p < 0.001). Subgroup analysis showed that this association was generally consistent across all subgroups, but there was significant heterogeneity by drinking status (P for interaction = 0.011). Specifically, the association was stronger in non‑drinkers (OR=2.94, p < 0.001) but not statistically significant in drinkers (OR=1.01, p = 0.974). No significant interactions were observed for other subgroups, including gender, marital status, economic status, smoking status, and residence (all P for interaction>0.05).

**Table 3:** Association between upper limb function and cognitive impairment: subgroup analyses (adjusted for all covariates).

Variables	n (%)	Normal cognition	Cognitive impairment	OR (95%CI)	P	P for interaction
All patients	5060 (100.00)	598/4590	157/470	2.55 (1.95-3.29)	<0.001	
**Gender**						0.373
Male	2154 (42.57)	335/1926	90/228	2.26 (1.54-3.31)	<0.001	
Female	2906 (57.43)	263/2664	67/242	2.80 (1.96-4.00)	<0.001	
**Marry**						0.690
Others	689 (13.62)	231/586	74/103	2.81 (1.61-4.92)	<0.001	
Married	4371 (86.38)	367/4004	83/367	2.51 (1.86-3.38)	<0.001	
**Economic Status**						0.129
Average and below	4017 (79.39)	511/3619	139/398	2.81 (2.12-3.72)	<0.001	
Affluent and above	1043 (20.61)	87/971	18/72	1.54 (0.75-3.15)	0.236	
**Smoke**						0.284
No	4075 (80.53)	513/3679	142/396	2.71 (2.05-3.59)	<0.001	
Yes	985 (19.47)	85/911	15/74	1.66 (0.79-3.49)	0.180	
**Drink**						0.011
No	4116 (81.34)	526/3724	141/392	2.94 (2.22-3.90)	<0.001	
Yes	944 (18.66)	72/866	16/78	1.01 (0.47-2.18)	0.974	
**Residence**						0.996
City	1316 (26.01)	104/1179	36/137	2.57 (1.46-4.54)	0.001	
Town	1612 (31.86)	211/1466	51/146	2.49 (1.59-3.90)	<0.001	
Rural	2132 (42.13)	283/1945	70/187	2.64 (1.77-3.93)	<0.001	

OR: Odds Ratio, CI: Confidence Interval

*Adjusted for age, gender, marital status, years of education, self-rated economic status, smoking, drinking, and residence.

As shown in Table 4, the association between physical exercise and cognitive impairment was generally consistent across subgroups, but there was significant heterogeneity by drinking status (P for interaction = 0.030). Specifically, the association was stronger in non‑drinkers. No significant interactions were observed for other subgroups (gender, marital status, economic status, smoking status, residence) (all P for interaction > 0.05).

**Table 4:** Association between physical exercise and cognitive impairment: subgroup analyses (adjusted for all covariates).

Variables	n (%)	Normal cognition	Cognitive impairment	OR (95%CI)	P	P for interaction
**All patients**	5060 (100.00)	592/3101	163/1959	0.77 (0.62-0.95)	0.014	
**Gender**						0.566
Male	2154 (42.57)	348/1413	77/741	0.88 (0.64-1.21)	0.415	
Female	2906 (57.43)	244/1688	86/1218	0.69 (0.52-0.92)	0.012	
**Marry**						0.560
Others	689 (13.62)	263/512	42/177	0.69 (0.43-1.10)	0.121	
Married	4371 (86.38)	329/2589	121/1782	0.79 (0.62-1.00)	0.052	
**Economic Status**						0.710
Average and below	4017 (79.39)	516/2565	134/1452	0.79 (0.62-0.99)	0.044	
Affluent and above	1043 (20.61)	76/536	29/507	0.70 (0.42-1.17)	0.178	
**Smoke**						0.073
No	4075 (80.53)	523/2484	132/1591	0.69 (0.55-0.87)	0.002	
Yes	985 (19.47)	69/617	31/368	1.27 (0.76-2.14)	0.359	
**Drink**						0.030
No	4116 (81.34)	536/2553	131/1563	0.69 (0.54-0.87)	0.002	
Yes	944 (18.66)	56/548	32/396	1.41 (0.83-2.41)	0.208	
**Residence**						0.064
City	1316 (26.01)	100/564	40/752	0.50 (0.32-0.79)	0.003	
Town	1612 (31.86)	208/1037	54/575	0.74 (0.52-1.06)	0.105	
Rural	2132 (42.13)	284/1500	69/632	0.99 (0.71-1.36)	0.936	

OR: Odds Ratio, CI: Confidence Interval

*Adjusted for age, gender, marital status, years of education, self-rated economic status, smoking, drinking, and residence.

Based on the study results regarding the association between upper limb function, physical exercise, and cognitive ability in Chinese empty-nest elderly, we conducted a sensitivity analysis to assess the robustness of these associations. We employed linear regression models with stepwise adjustment for confounders. As shown in Table 5, these significant associations remained stable across all models. Specifically, restricted upper limb function was consistently associated with an increased risk of cognitive impairment (β = −3.60, 95% CI: −4.13--3.07, p < 0.001). Higher frequency of physical exercise was associated with a decreased risk of cognitive impairment (β = 2.10, 95% CI: 1.78–2.41, p < 0.001). Significant associations were present from Model 1 to Model 3, further strengthening the robustness of the main findings.

**Table 5:** Association of upper limb function and physical exercise with cognitive function using linear regression models.

	β	95%CI	p
**Upper Limb Function**			
Model 1	−3.60	−4.13--3.07	<0.001
Model 2	−2.24	−2.69--1.79	<0.001
Model 3	−2.25	−2.69--1.80	<0.001
**Exercise**			
Model 1	2.10	1.78-2.41	<0.001
Model 2	0.74	0.47-1.02	<0.001
Model 3	0.74	0.46-1.02	<0.001

## Discussion

This study explored the associations among upper limb function, physical exercise, and cognitive ability in Chinese empty-nest elderly based on a nationally representative sample. Our findings reveal that restricted upper limb function is significantly associated with an increased risk of cognitive impairment, and physical exercise was found to play a partial mediating role in this relationship.

This study found that individuals with restricted upper limb function had a significantly higher risk of cognitive impairment compared to those with normal function. The results of this study are consistent with existing literature, indicating that changes in upper limb motor parameters, such as slowed speed, increased errors, and greater variability, are closely associated with cognitive impairment. Particularly in cognitive-motor dual-task paradigms, upper limb motor performance can effectively discriminate patients with mild cognitive impairment, further reinforcing its clinical utility [9]. The mechanism underlying this association may stem from shared neural substrates. Complex upper limb movements, especially dexterous hand activities, highly depend on intact cognitive domains such as executive function and attention [13]. Cognitive impairment may lead to reduced efficiency in motor learning processes and motor planning, directly manifesting as restricted upper limb function. Conversely, studies have shown that cognitive reserve may buffer the degree of motor impairment by modulating functional connectivity within brain networks, revealing the complex interaction between cognitive and motor functions [14]. From prognostic and clinical management perspectives, upper limb motor function is not only a status indicator but also a valuable predictor. Prospective studies confirm that performance on functional upper limb tasks can predict future cognitive decline and deterioration in daily activity abilities [15]. For example, long-term deterioration in grip strength and manual dexterity has been identified as a risk marker for cognitive decline [16,17]. For populations such as stroke survivors, the compounding of cognitive impairment and upper limb motor impairment further limits the use of the affected limb and independence in daily activities, creating a vicious cycle of functional decline [18]. In summary, the findings of this study reinforce the necessity of integrating upper limb function assessment into routine clinical practice for the elderly population. Simple upper limb function tests, such as grip strength tests or manual dexterity tasks, can serve as low-cost, non-invasive tools for early identification of individuals at risk for cognitive decline and for tracking disease progression. Future research should focus on standardizing assessment protocols for upper limb motor function and further exploring the efficacy of targeted upper limb exercise interventions (such as fine motor training or dual-task training) in delaying cognitive decline and improving overall functional outcomes.

Physical exercise is widely recognized as a key strategy for maintaining and enhancing cognitive function in older adults. The results of this study are consistent with a substantial body of existing evidence, reinforcing the broad positive impact of physical exercise on cognitive ability in the elderly. For instance, the study by Sewell Kelsey R. et al. indicated that physical exercise can improve cognitive ability and delay the onset of dementia [19]. Blomstrand, P. et al., through a meta-analysis, summarized the effects of physical exercise on cognitive function (global cognition, executive function, memory, attention, or processing speed) in healthy adults aged ≥55 years, showing that mind-body exercise has a moderately positive effect on cognitive function in individuals aged 55 and above [20]. Notably, the effects of physical exercise vary across different cognitive domains. For executive function—a higher-order cognitive ability involving planning, decision-making, and multitasking—combined intervention programs incorporating aerobic exercise with resistance training show particularly significant effects, especially in very old populations [21]. For improving memory function, mind-body exercises (such as Tai Chi and yoga) demonstrate unique advantages, possibly due to their comprehensive effects on stress relief, attention improvement, and mind-body coordination [22]. However, the cognitive benefits of physical exercise vary across different populations. Its cognitive protective effect is clear for healthy older adults; but for individuals with mild cognitive impairment or dementia, the effect of a single exercise intervention may be limited [23]. Furthermore, exercise can also significantly improve self-reported cognitive function and executive function in special populations such as breast cancer survivors and cardiovascular disease patients, highlighting the important value of exercise in clinical rehabilitation [24,25]. Nonetheless, we must also acknowledge the limitations present in current research. The study by Iso-Markku, P. et al. suggested that the association between exercise and cognition is weak, particularly in the exercise-only intervention group [26]. These inconsistent results may be influenced by differences in sample characteristics (e.g., age, baseline cognitive level) and training parameters (e.g., intensity, frequency, total duration) [27]. Therefore, future research needs to focus on developing more personalized and precise exercise prescriptions and further elucidating the underlying neurobiological mechanisms through which they exert cognitive protective effects.

Our study found that physical exercise partially mediated the association between upper limb function and cognitive ability. Although the mediation proportion was small (5.59%), it was statistically significant. This result suggests that upper limb function may affect cognition both directly and indirectly through physical exercise. However, due to the cross‑sectional design, we cannot determine the temporal sequence or causal direction of these relationships. Upper limb function serves as one of the assessment indicators for sarcopenia. The study by Yao, X. et al. showed that physical activity plays a partial mediating role (20.2% effect size) between sarcopenia and cognitive dysfunction [28]. This means that sarcopenia not only directly affects cognitive function but also indirectly leads to cognitive decline by reducing physical activity levels, while physical exercise can buffer this negative impact. The relatively low mediation proportion in this study may stem from differences in population characteristics or assessment methods, but overall supports physical exercise as an important pathway in the association between upper limb function and cognition. Regarding the type of physical exercise, relevant studies indicate that targeted upper limb training (whether combined with lower limb training or not) can significantly improve executive function performance, with beneficial effects observed on tasks involving cognitive conflict (e.g., Stroop task) [29]. In studies on specific populations, hand and upper limb dexterity training can simultaneously improve cognitive function, upper limb function, and activities of daily living (ADL), which is particularly applicable to patients with cerebral ischemic stroke [30]. The research by Sanchez-Lastra, M. A. et al. further suggests that upper limb training may be more directly associated with cognitive function improvement compared to lower limb training, especially among institutionalized older adults, where the promoting effect of upper limb training on cognition is more prominent [31]. Therefore, future health interventions for empty-nest elderly should focus on integrating upper limb function exercises with overall physical activity promotion to delay cognitive decline through multiple pathways.

Subgroup analyses revealed differences in the associations of upper limb function and physical exercise with cognitive impairment across certain population strata. Specifically, the positive association between restricted upper limb function and cognitive impairment was stronger in non‑drinkers but not statistically significant in drinkers, with a significant interaction. Likewise, the protective effect of physical exercise against cognitive impairment was stronger in non‑drinkers, while no significant association was observed in drinkers. These findings suggest that drinking behavior may modify the relationships among upper limb function, exercise, and cognition. One possible explanation is that drinkers may have different health status or levels of social engagement, which could weaken the protective effect of physical exercise or mask the impact of functional limitations. Alternatively, the lack of association in these subgroups might be due to a “the healthy worker survivor effect” [32]. Older adults who continue to drink are a highly selected group with better baseline health or greater functional reserve. However, given the cross-sectional design, these subgroup findings should be considered hypothesis-generating rather than conclusive. Future longitudinal studies are needed to clarify whether drinking modifies the long-term cognitive benefits of physical exercise or the impact of upper limb dysfunction.

Of note, our sample comprised community-dwelling empty-nest elderly who were able to participate in the Chinese Longitudinal Healthy Longevity Survey. This raises the possibility that individuals with both upper limb function restriction and cognitive impairment may still be able to live independently. First, many of these older adults may receive informal care from spouses, neighbors, or relatives, even if they do not live with their children. Second, community support services in China, such as day care centers, home-based care services, and community health stations, may provide assistance with activities of daily living. Third, environmental adaptations (e.g., modified utensils, handrails, simplified clothing) may enable some individuals to maintain a degree of independent function despite motor and cognitive deficits. Nevertheless, the cross‑sectional nature of the data prevents us from determining whether individuals with more severe conditions were systematically undercounted because they had been placed in institutions or did not participate in the survey. Future longitudinal studies should follow this population more closely to understand the dynamic interactions among functional limitations, support systems, and the ability to age at home.

Although this study, through large-sample data, revealed the associations among upper limb function, physical exercise, and cognitive ability in Chinese empty-nest elderly and verified the mediating role of physical exercise, several limitations remain: First, this study used cross-sectional data. Although significant associations and mediating effects between variables were found, causality cannot be established. Bidirectional influences may exist among upper limb function, physical exercise, and cognitive ability, requiring further verification through longitudinal studies or intervention experiments. Second, potential bias in self-reported data: Information on upper limb function and physical exercise primarily relied on self-reports, which may be subject to recall bias or social desirability bias. Future studies using continuous objective measures (e.g., grip strength, motion capture) could provide more nuanced assessments of upper limb function. Third, other uncontrolled confounding factors: Although variables such as gender, marital status, economic status, smoking, and drinking were included in the models, there may still be unobserved confounding factors (e.g., genetic background, dietary habits, social participation level) affecting the interpretability of the results. Fourth, limited sample representativeness: Although the study sample covered urban, town, and rural areas, the sampling method was not completely random. The proportion of certain subgroups (e.g., smokers, drinkers) in the sample was relatively low, which may affect the stability and generalizability of the subgroup analysis results.

## Conclusion

We observed that restricted upper limb function was positively associated with cognitive impairment, whereas regular physical exercise was negatively associated. Although physical exercise accounted for a small (5.95%) and statistically significant proportion of the association, this mediating pathway should not be overemphasized as a primary mechanism. Other unmeasured factors-for example, vascular health, neuroinflammation, and social engagement-may have more substantial influences. Future research using longitudinal designs is needed to test these possibilities.

## Supporting information

S1 Table Comparison of baseline characteristics between included and excluded participants. This table presents the comparison of demographic characteristics, health behaviors, and functional status between the final analytical sample (n = 5,060) and participants excluded due to missing data on key variables (n = 2,654). No statistically significant differences were observed for most characteristics (P > 0.05).
(PDF)
