# Supplementary material for: The association between upper limb function, physical exercise, and cognitive ability among empty-nest elderly in China: A cross-sectional study based on CLHLS
Source: PLoS One. 2026 Jun 10;21(6):e0351211. doi: 10.1371/journal.pone.0351211 (PMC13252726; doi:10.1371/journal.pone.0351211)
Supplement: S1 Table — This table presents the comparison of demographic characteristics, health behaviors, and functional status between the final analytical sample (n = 5,060) and participants excluded due to missing data on key variables (n = 2,654). No statistically significant differences were observed for most characteristics (P > 0.05). (PDF) [file pone.0351211.s001.pdf]

S1 Table. Comparison of baseline characteristics between included and excluded participants.

Variables	Total (n=7714)	Excluded (n=2654)	Included (n=5060)	*P*
**Age, Mean±SD**	78.76±10.35	79.08±10.40	78.60±10.31	0.055
**Upper Limb Function, Mean±SD**	11.81±0.67	11.80±0.68	11.81±0.67	0.538
**Years of Education, Mean±SD**	4.69±4.69	4.60±4.70	4.74±4.69	0.217
**Mmse, Mean±SD**	26.65±5.68	26.49±5.70	26.73±5.67	0.079
**Sex, n(%)**			n=5060	0.082
Male	3319 (43.03)	1165 (43.90)	2154 (42.57)	
Female	4351 (56.40)	1445 (54.45)	2906 (57.43)	
**Marital, n(%)**				0.450
Others	1059 (13.73)	370 (13.94)	689 (13.62)	
Married	6596 (85.51)	2225 (83.84)	4371 (86.38)	
**Econ State, n(%)**				0.868
Average and below	6081 (78.83)	2064 (77.77)	4017 (79.39)	
Affluent and above	1584 (20.53)	541 (20.38)	1043 (20.61)	
**Smoke, n(%)**				0.202
No	6165 (79.91)	2090 (78.74)	4075 (80.53)	
Yes	1530 (19.83)	545 (20.54)	985 (19.47)	
**Drink, n(%)**				0.530
No	6236 (80.84)	2120 (79.88)	4116 (81.34)	
Yes	1449 (18.78)	505 (19.03)	944 (18.66)	
**Exercise, n(%)**				0.763
No	4713 (61.10)	1612 (60.74)	3101 (61.28)	
Yes	2962 (38.40)	1003 (37.79)	1959 (38.72)	
